# Towards More Precise Targeting of Inhaled Aerosols to Different Areas of the Respiratory System

**DOI:** 10.3390/pharmaceutics16010097

**Published:** 2024-01-10

**Authors:** Tomasz R. Sosnowski

**Affiliations:** Faculty of Chemical and Process Engineering, Warsaw University of Technology, Waryńskiego 1, 00-645 Warsaw, Poland; tomasz.sosnowski@pw.edu.pl

**Keywords:** aerosol, nebulization, inhalation, airflow dynamics, particle deposition, particle–lung interactions, smart inhalers, AI in drug delivery

## Abstract

Pharmaceutical aerosols play a key role in the treatment of lung disorders, but also systemic diseases, due to their ability to target specific areas of the respiratory system (RS). This article focuses on identifying and clarifying the influence of various factors involved in the generation of aerosol micro- and nanoparticles on their regional distribution and deposition in the RS. Attention is given to the importance of process parameters during the aerosolization of liquids or powders and the role of aerosol flow dynamics in the RS. The interaction of deposited particles with the fluid environment of the lung is also pointed out as an important step in the mass transfer of the drug to the RS surface. The analysis presented highlights the technical aspects of preparing the precursors to ensure that the properties of the aerosol are suitable for a given therapeutic target. Through an analysis of existing technical limitations, selected strategies aimed at enhancing the effectiveness of targeted aerosol delivery to the RS have been identified and presented. These strategies also include the use of smart inhaling devices and systems with built-in AI algorithms.

## 1. Introduction

Aerosol particles are convenient carriers of therapeutic agents targeted to the respiratory system (RS). This method of drug delivery has been known since ancient times, where inhaling smoke from burning plant leaves was considered to relieve respiratory ailments [1,2]. In modern times, dedicated medical devices for aerosol generation for inhalation purposes (nebulizers and inhalers) have been developed. Also, the beneficial effects of inhaling saline aerosols/salt particles from the environment have contributed to the development of halotherapy as a method of pulmonary rehabilitation [3], which can be also carried out under specific conditions of underground treatment in caves and salt mines (subterranean therapy) [4,5]. In addition to treating respiratory diseases, aerosols can also carry pharmaceuticals absorbed into the circulation and acting in other organs (systemic drugs). This can include insulin [6,7], painkillers [8], neuroactive substances [9,10], and even cardiovascular drugs [11], confirming that inhalation has the potential for applications in new therapeutic areas.

Despite the apparently easy (natural) way to introduce aerosol into the lungs via inhalation, obtaining particles with optimal properties and applying them to maximize their delivery to specific areas of the RS is challenging [12,13]. Among the important elements responsible for this are the complex geometric structure of the RS in which the aerosol must be transported and distributed, and the physics of aerosols during their actual airflow in the body. This paper highlights the most important, albeit often overlooked, factors responsible for successful aerosol drug targeting in the RS, and attempts to identify and indicate new opportunities to improve this therapeutic method.

## 2. Major Factors Influencing the Efficiency of Drug Delivery Using Aerosols

### 2.1. Basic Concepts of Inhalation Therapy

Aerosol is a thermodynamically unstable system, so it cannot be prepared in advance and stored until use and must be generated just before inhalation. This is achieved with the help of special, sometimes complicated, technical devices (inhalers or nebulizers). Since neither aerosol generation nor aerosol deposition is fully effective (efficiency below 100%), the amount of drug delivered to the target area that should induce the expected therapeutic effects is always lower than the initial dose loaded into the aerosolizing device [14,15]. 

Different physical mechanisms act on inhaled particles with various properties (e.g., shape and density) and sizes (micro- and nanoparticles), influencing the efficiency of their delivery to different parts of RS [15]. These mechanisms also depend on the actual flow in the system, and it is important to note that respiration is inherently associated with unstable (oscillatory) aerodynamic conditions, which strongly affect the behavior of aerosol particles. In addition, aerosol particles can also change their size during their lifetime, e.g., due to coagulation, condensation, or evaporation (if they are liquid droplets). 

**Figure 1:**
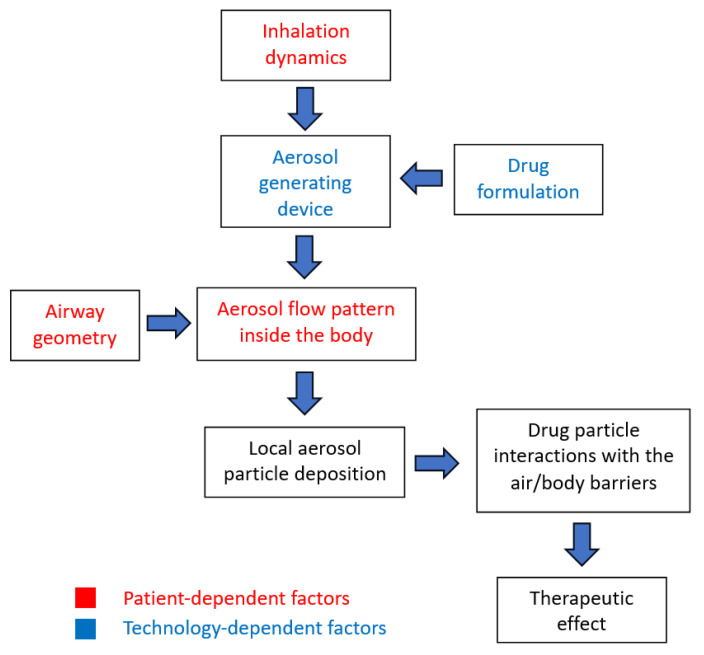
The basic processes and factors shaping the efficiency of drug delivery using aerosols.

According to the above, it is clear that—unlike many other drug delivery systems (injections, capsules and tablets, and suppositories)—the dose of inhaled aerosol drug delivered to the target areas cannot be precisely known, at it depends on many patient- and technology-related factors, which are indicated in Figure 1. Inhalation dynamics and airway geometry are individual patient characteristics and determine the flow of air or aerosol through the airways. However, a medical aerosol is generated from a specific precursor using an airflow through an inhaler. In this process, both the precursor and the inhalation device are optimized using an engineering approach (i.e., technology). As the aerosol is carried into the respiratory tract by the flow produced by the patient, the factors related to both the patient and the technology are combined, leading to deposition of the drug in different parts of the RS (local deposition). The drug delivered to the surface of the RS will act effectively when it overcomes the barrier formed by the fluids covering the airways, meaning that the interactions between the deposited particles and the surface of the RS are the final step in targeting the drug to different parts of the respiratory system.

### 2.2. Inhalation Dynamics and Particle Deposition Mechanisms

A considerable number of studies on the experimental and numerical modeling of aerosol flow and deposition in the human respiratory tract are available in the literature [16,17,18], so only selected, sometimes underestimated or less frequently discussed issues will be highlighted here. 

**Figure 2:**
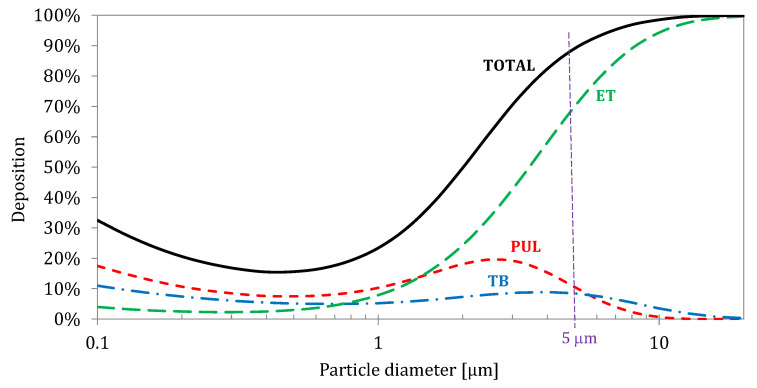
Regional deposition (expressed as % of inhaled particles) of aerosol delivered via mouth as a function of particle size (aerodynamic diameter) for normal breathing: ET—extrathoracic deposition; TB—tracheobronchial deposition; and PUL—pulmonary deposition. Data calculated according to Multiple-Path Particle Dosimetry Model (MPPD) model for Yeh–Schum symmetric bronchial geometry [20].

Upon inhalation, aerosol passes the airways of the head region (nasal or oral cavity, and throat) and then it penetrates through the geometrically complex tracheobronchial tree. Inhaled particles undergo selective separation (deposition) in subsequent areas of the RS through which they flow. This explains why large particles cannot be used to effectively target the pulmonary region. The schematic deposition efficiency curve for inhaled aerosol particles in the size range of 0.1–15 μm, as the most important for the inhalation therapy [19], is presented in Figure 2 [20]. These data also show that the (for any particle size) fraction of inhaled drug is always deposited in the regions outside this target, which can raise some concerns regarding the safety of the inhalation therapy, i.e., side effects [21]. It should also be noted that some particles (mainly in the size range of 0.2–0.5 μm) are not deposited and are exhaled, since all deposition mechanisms for this size are ineffective. 

**Figure 3:**
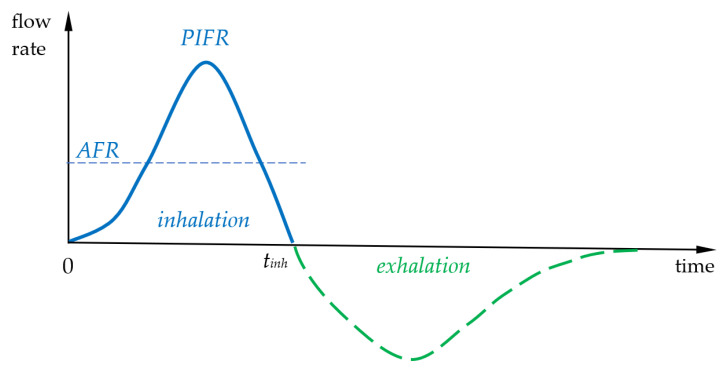
Schematic airflow variation vs. time during inhalation and exhalation. *AFR*—average flow rate during inhalation; *PIFR*—peak inspiratory flow rate; and *t_inh_*—time of inhalation.

Most inhaled particles are deposited during aerosol entry into the lungs (inhalation), but some are also deposited during breath-holding [22,23] (which is often recommended as a good practice for patients using inhalers) as well as exhalation. Typical airflow during unforced inhalation (at rest) which is used, e.g., during nebulization, is characterized by the frequency (breathing rate, *BR*) of 12–15 min^−1^ and total inhaled volume per breath (tidal volume, *TV*) of 500 mL. It can be calculated that the average inhalation flow rate (*AFR*) is 200–250 mL/s, i.e., 12–15 L/min, whereas the peak inspiratory flow rate (*PIFR*) for tidal breathing is 20–60 L/min or higher [24,25,26], as seen in Figure 3.

From any type of inhalation curve, the *AFR* can be calculated according to

(1) $$AFR=\frac{TV}{t_{inh}}=\frac{1}{t_{inh}}\;\int_{0}^{t_{inh}}Q(t)dt$$

where *t_inh_* is the inhalation time and *Q(t)* is the instantaneous airflow rate. As seen in Figure 3, the shape of the exhalation curve is slightly different, especially in obstructive lung diseases when the duration of air outflow is extended, but, of course, all previously inhaled volume is forced out.

As already mentioned, particles are deposited in the RS via several mechanisms, mainly impaction, gravitational sedimentation, interception, and Brownian diffusion [15,16]. Both the properties of the aerosol particles (mainly size and shape) and the temporal variation in air velocity affect these deposition mechanisms, making it difficult to precisely determine the clear relationship between, for example, particle diameter and its deposition efficiency in the given airways at each moment of inhalation. The common misunderstanding is assuming that particles of a given size can be deposited only in a certain region or even in a single bronchial generation. Even particles with one size (monodisperse aerosol) show a spatial distribution of deposition in the RS. This can be easily seen in Figure 2, which shows that the deposition of 5 μm particles takes place mainly in the extrathoracic region (approx. 70% of the inhaled amount), but also in the tracheobronchial (approx. 9%) and the pulmonary regions (approx. 11%). 

Theoretically, the behavior of aerosol particles in the RS can be analyzed using dimensionless numbers which are typically applied in the characterization of the airflow and aerodynamic forces acting on aerosol particles in other (mainly technical) applications [27]. However, it is important to be aware of some limitations of using common criteria such as Reynolds numbers (Re) and Stokes numbers (Stk), since the air velocity in the system is variable, and, at the same time, the airways of a certain diameter are short, leading to a strong influence of inlet (entrance) effects on the airflow field [28]. For instance, the airflow through the trachea during breathing changes from laminar at the start of inhalation to turbulent at *PIFR*, and then it becomes laminar again when the flow decreases down to zero (see Figure 3) [29]. 

**Figure 4:**
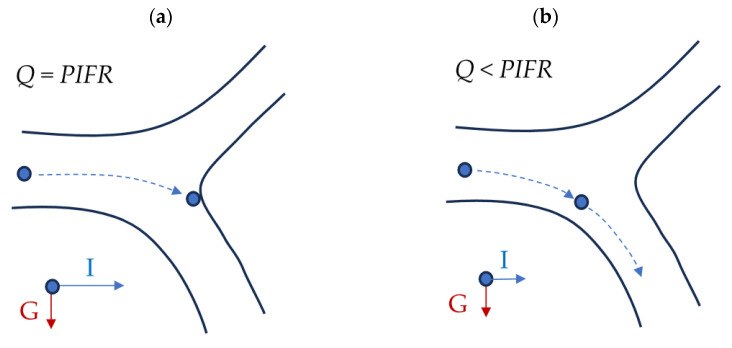
Schematic trajectory of a few-micrometer particle moving through a bronchial bifurcation at different time instants of inhalation and the corresponding different values of the flow rate *Q:* (**a**) *Q* equal the peak inspiratory flow rate (*PIFR*), or (**b)** *Q* smaller than *PIFR.* Blue and red arrows represent the instantaneous forces acting on the particle due to inertia (I) and gravitation (G).

The variations in airflow structure influence particle fate in the airways. For example, if 5 μm particles are deposited by impaction in a certain generation at *PIFR*, it may be expected that such particles can avoid inertial deposition in the same generation when the flow is slower, i.e., at all other moments of inhalation, because the inertial force acting on such particles will be weaker (Figure 4). On the other hand, slower flows increase the probability of deposition by sedimentation (for large particles) and diffusion (for particles smaller than 0.1 μm). This is due to a longer residence time of the aerosol in a given element of RS, i.e., longer time available for particle landing on the airway wall due to gravitation. This analysis shows that there is an interplay between particle size and its local and temporal velocity in the airways. 

It follows that the common assumption of steady flow which is used in many CFD and experimental in vitro studies is unrealistic and may result in unprecise understanding and predictions of drug delivery to different regions of RS. For example, an analysis by Sosnowski et al. [26] showed that the actual airflow structure inside the oral cavity and pharynx at any phase of inspiration is never equal to the structure representing the average flow. This was confirmed also for bronchial airways [29,30]. The results of particle deposition in a given location inside the airways which are obtained assuming *Q* = *AFR* also differ from the values obtained from summing the instantaneous deposition during the cycle, and this can be easily explained by the fact that the local deposition efficiency of particles with a certain size is not a linear function of the flow rate. Therefore, the cumulative number (or mass) of drug particles deposited in each RS region during inhalation cannot be assessed by evaluating the flux for the *AFR* and extrapolating it over the entire time *t_inh_* [31,32,33].

The assumption of the steady-state flow also neglects all transient effects, which influence particle deposition. Secondary flows, which are formed during realistic breathing in the regions of bronchial bifurcation, can be characterized by Womersley number, Wo:

(2) $$Wo=R\sqrt{\frac{\omega }{\nu }}$$

where *R* is radius of the tube (bronchus), *ω* is the angular frequency of breathing, and *ν* is the kinematic viscosity of air. These were shown to increase the deposition of inhaled particles [29,30,31,32]. Another common simplification in many CFD studies is the assumption of a parabolic profile at the inlet the trachea, which neglects airflow disturbances generated before the air enters the bronchial tree (laryngeal jet) [34,35].

It should be also remembered that particles which have entered the respiratory system during inhalation but were not deposited during this period can be deposited during other phases of breathing. In particular, the instant of stopping and the reversal of flow direction from inhalation to exhalation can contribute to the enhanced deposition of extra-fine particles [29,33,36]. 

### 2.3. The Influence of Airway Geometry

**Figure 5:**
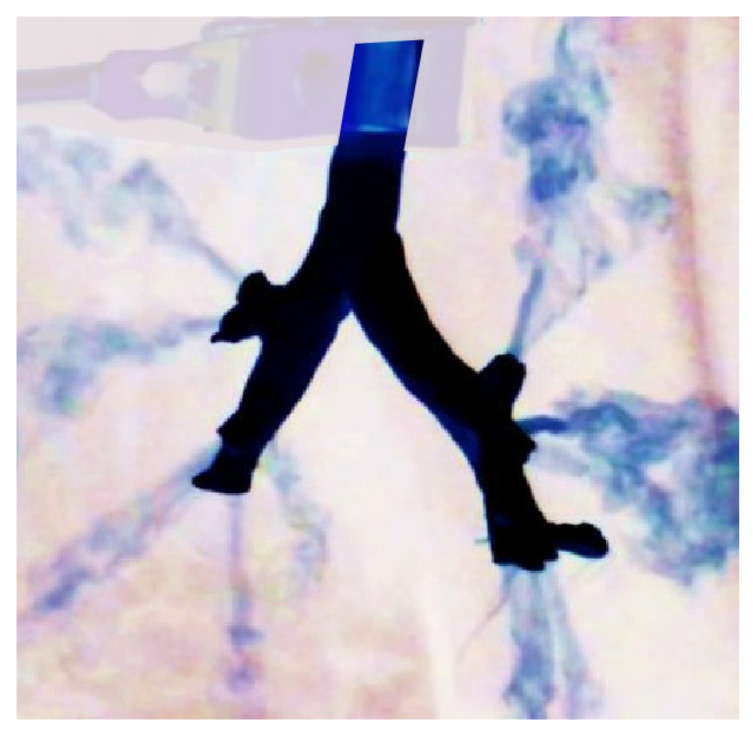
Visualization of aerosol penetration through reconstructed large airways of COPD patient during simulated inhalation. Bronchial geometry according to [37].

Almost all quantitative deposition data available in the literature, obtained with in vivo, in vitro, and in silico (CFD—computational fluid dynamics) studies, are obtained for healthy subjects. This is partly because many results were obtained in the field of inhalation toxicology and then adapted to inhalation therapy. However, inhalation drugs are administered to patients suffering from lung diseases such as asthma, chronic obstructive pulmonary disease (COPD), cystic fibrosis, pulmonary hypertension, etc. In all these cases, the airways are locally narrowed or obstructed due to excessive mucus secretion and inflammation. This changes the geometry of bronchial airways, resulting in different airflow and different distributions of particle deposition. Kadota et al. [37] compared the results of CFD calculations for three actual geometries of COPD patients, showing the dependence of drug delivery effectiveness on disease severity. The results obtained in our laboratory show the variable distribution of aerosol flow through the bronchi of reconstructed 3D bronchial geometry of a patient with COPD according to data from [37], as shown in Figure 5. The observed flow structure will affect the mass of aerosol that penetrates to different areas of the lungs, as predicted by CFD computations.

## 3. The Role of the Aerosol-Generating Device and the Form of the Precursor

It was already highlighted that an aerosol must be generated directly before inhalation due its intrinsic instability [38]. Even if the time scale required for the use of freshly produced aerosol is short, rapid changes in the aerosol properties may lead to its inapplicability to a given therapeutic purpose. The method of aerosol generation is dictated by the form of the precursor, which can be either liquid (solution or suspension) or solid (powder). 

Medicinal liquids are atomized into fine droplets using several methods: by applying pressure to the liquid forced through the nozzle, pneumatically (using compressed air), via ultrasounds [39], via the collision of liquid streams [40], electro-hydrodynamically [41], using surface acoustic waves [42], etc. The size distribution of generated liquid droplets can be adjusted to certain therapeutic applications by the design of the atomizing device, process parameters, and liquid properties. 

Aerosol generation from powders must combine powder fluidization (transfer to the gas phase) with particle deagglomeration (break-up of particle clusters) [43]. This requires turbulent flow through the inhaler, and is usually related to a high pressure drop which must be overcome by the patient during inhalation. This is why a special inspiration maneuver is always required when using dry powder inhalers (DPIs) to assure a forceful flow with a defined duration. It should be noted that the required flow pattern is not always achievable with certain DPIs, since patients are of different ages and health conditions (e.g., children, elderly, and patients with COPD); therefore, they have different inhalation force and capability to inhale according to the requirements. The emitted dose and particle size distribution of the aerosol particles can be flow-dependent, and they can affect the DPI design and drug formulation [44].

The preparation of powders with the properties required to obtain aerosols using DPIs suitable for targeted drug delivery is another challenge. Of course, the powder grains must be smaller than the expected aerosol particle size, but this does not guarantee that all aerosol particles will be small enough, since micrometer-sized particles easily form permanent agglomerates [45]. This is why inhalable powders are typically prepared as a mixture of fine grains of a drug with larger particles of an excipient (usually lactose). Such blends are more easily fluidized and deagglomerated than fine grains without the excipient. However, the preparation of blends is also demanding, especially for drugs which contain more than one therapeutic substance [46]. Some powders can be engineered in a way to allow them to be aerosolized without lactose [47,48,49].

In contrast to liquid droplets, engineered powder particles can be of a different shape and can also be porous, which can be used to maximize their local delivery to different regions of the RS. The programmed non-sphericity of particles can be used to obtain better penetration, deposition, and functionality of inhaled drugs [50,51,52].

The above discussion shows that the optimal aerosol properties are defined by the target of drug delivery which, therefore, also influences the choice of method/device for generating particles of a given size range. Below, two targets and the corresponding drug delivery devices will be presented.

### 3.1. Drug Targeting to the Lower Respiratory System

Several types of inhalation devices are typically used to generate and target medical aerosol to the lower RS. The three most used are pressurized metered dose inhalers (pMDIs), dry powder inhalers (DPIs), and nebulizers, but other designs of inhalers are also present on the market (Table 1). Each class of these devices can be subdivided into additional subtypes. Medical aerosols targeted to the RS should be inhaled through the mouth to reduce the deposition of the drug in the upper airways (extrathoracic region). However, aerosolized therapeutics are also delivered to the lungs through the nose using inhalation masks, which are useful when aerosol administration through the mouthpiece is not possible or ineffective (e.g., small children, and disabled or uncooperative patients). Transnasal administration can also be performed during oxygen supplementation using a high-flow nasal cannula (HFNC), when oxygen-enriched air is used to carry the aerosol which is preferably delivered from a nebulizer with a low internal flow, such as VMN [53,54].

Only nebulizers and HFNC systems allow patients to inhale aerosol with spontaneous (i.e., not forced) breathing, which makes these inhaling devices the most versatile Ingelhein Boej [58,59]. In other cases, i.e., when the inhalation is restricted by the external resistance of the inhaler and requires the additional inspiratory effort of a patient, the flow function *Q(t)* (also: *PIFR* and *AFR*—see Figure 2) will be different. During inhalation, the lungs must adapt to the inhaling device [60,61]. This process can be understood as achieving the duty point of the pump, where the actual flow rate *Q* and the corresponding pressure drop in the system, Δ*P*, depend both on the pump capacity (here, lung capacity) and the external resistance (here, the inhaler). In other words, the actual airflow rate depends on the internal aerodynamic resistance of the inhaler, as the mechanical capacity of the lungs is only partly adjustable to the flow restrictions [62]. This shows that the frequently used PIFR value, which is measured under arbitrary conditions, can be misleading because it is not obtained with all inhalers with different resistances. It is especially important in DPIs, where the air must be drawn forcefully through the device to assure the required powder aerosolization [60,63].

DPIs are classified into low-, medium-, and high-resistant devices based on the value of intrinsic aerodynamic resistance *R_D_*, defined as

(3) $$R_{D}=\frac{\sqrt{\Delta P}}{Q}$$

and some high-resistant DPIs are not appropriate for patients with a compromised lung mechanics. Also, for pMDIs, patients should adapt to the special breathing maneuver, i.e., slow and deep inhalation followed by a breath-hold [14]. Therefore, the inhalation curve shown schematically in Figure 3 is applicable only to nebulizers, which have low resistance and can be used with spontaneous breathing.

### 3.2. Drug Targeting to the Nasal Cavity

The topical delivery of medicines to the surface of the nasal cavity is important since almost a quarter of the population suffers from allergic rhinitis [64], requiring the use of locally acting decongestants and anti-inflammatory drugs. These medicines in the form of nasal drops are not comfortable to use and often run down the throat, causing irritation. Aerosols appear more convenient and effective in the homogeneous delivery of drugs to the nasal cavity. However, due to the narrow and tortuous nasal air passages, delivering medication to this region is difficult.

**Figure 6:**
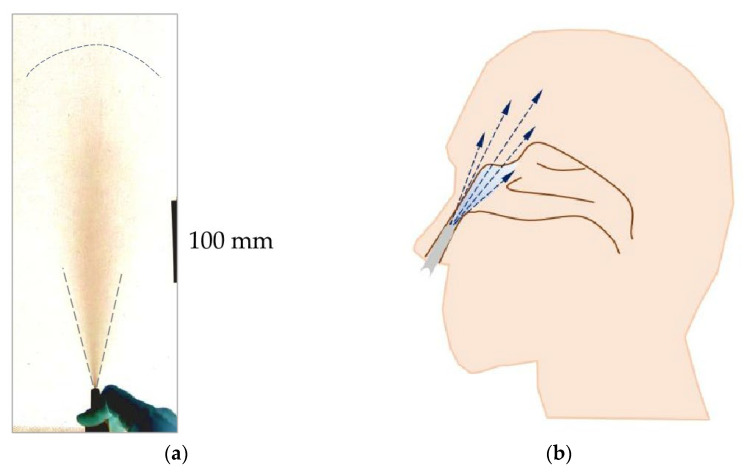
(**a**). An aerosol plume obtained with the manually actuated nasal atomizer (dashed lines schematically show the limits of the expanding plume). (**b**). Schematically drawn incompatibility of the expanding aerosol plume and narrow nasal airways, resulting in predominant anterior deposition of sprayed liquid (based on results published by Sosnowski et al. [65]).

Drug delivery to the surface of the nasal cavity requires relatively large aerosol particles (30–80 μm) to avoid their penetration into the throat and lower RS. The generation of such aerosol can be easily achieved from liquids via atomization in hand-operated nasal pumps. The pressure applied on the liquid is low but is enough to produce droplets of the required size. However, as in all atomization processes, a small volume of liquid is converted to a large volume of a gas–liquid system, i.e., aerosol. A well-dispersed aerosol plume of droplets ejected from the atomizer nozzle is up to hundreds of millimeters in length and up to 100 mm in width, depending on the liquid properties and nozzle design [65,66], which makes it incompatible with nasal geometry, as shown in Figure 6. Therefore, after placing the atomizer tip inside the nostril, the plume cannot evolve, and the ejected liquid is deposited on the nearby surfaces of the nasal sidewall and nasal septum [65]. Under such conditions, an even drug distribution across the whole nasal cavity is not possible. This situation is different compared to the free penetration through the nasal geometry of aerosol inhaled from nebulizers. Since drugs aerosolized in nasal pumps cannot be directly delivered to all parts of the nasal cavity, the pharmaceutical effects observed after drug application need additional explanation. This can be provided by the gravitational runoff of deposited liquid, as well as liquid spreading to deeper parts of the nose caused by the aerodynamic interactions between the inhaled air and the liquid layer of drug deposited in the front of the nose [65,67]. The kinetics of such a translocation of the liquid drug depend on its rheological properties [68], the deposited volume, and probably also on other factors such as wetting/adhesion forces. It was also confirmed that the high velocity of airflow, caused by intense drawing of the air through the nose in a natural reflex to prevent drug drainage, significantly improves the spread of the drug into the deeper regions of the nasal cavity [65].

The intranasal administration of drugs as aerosols becomes more difficult in cases of airway obstruction caused by anatomical abnormalities (obturation, deviated nasal septum, and polyps). In such situations, aerosols administered as relatively large droplets sprayed from a nasal pump are even less effective. Some concepts of using acoustic vibrations have been proposed to enhance aerosol penetration in congested or partly blocked nasal airways [69]. In this application, the aerosol droplets are finer, which allows them to be carried with the air along the nasal air passages. Due to the additional pressure wave, they are deposited on the walls of the nose and do not penetrate to the deeper structures of RS [70,71].

## 4. Particle–Lung Interaction and the Mass Transfer after Drug Deposition

**Figure 7:**
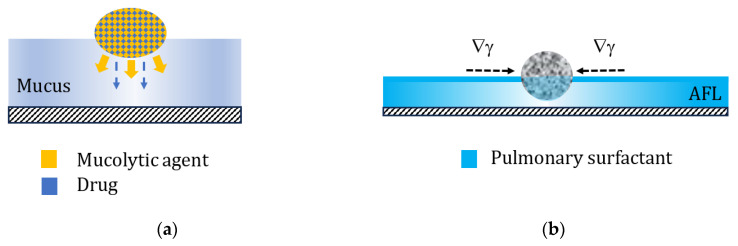
(**a**). Schematic action of the composite drug–mucolytic particle in the bronchial mucus: mucus-thinning component (yellow) accelerates diffusion of the drug (blue) through the liquid layer. (**b**). Local reduction in the concentration of PS on the alveolar surface due to surfactant adsorption onto a porous particle. AFL—alveolar fluid layer; ∇γ—gradient of the surface tension caused by adsorption of surfactant molecules on the particle according to the concept proposed in [80].

The process of delivering an aerosol drug involves its deposition on the surface of the respiratory system and its subsequent interaction with this surface [72]. The surface of RS is covered by fluids that act as a protective barrier function, and these are mucus in the bronchial tree or pulmonary surfactant in the alveolar region. The composition and physicochemical properties of these fluids very often determine the bioavailability or safety of a given drug, but also help to develop the most favorable formulation. These may include, for instance, multicomponent composite particles, where one component (mucolytic agent, e.g., N-acetylcysteine) interacts with viscous mucus, causing its local thinning and facilitating the diffusion of the second (therapeutic) component [73,74], or particles with mucoadhesive properties that extend the residence time of the drug on the bronchial surface [75]. Many drug particles are soluble in the liquid covering the lung surface; however, the use of poorly soluble engineered particles (e.g., porous and nanostructured) can also be considered attractive. The porosity of the particles allows us to obtain the effects of a controlled reduction in the natural defense mechanisms on the alveolar level, prolonging the residence time of particles deposited in this area. This action is related to the presence of the pulmonary surfactant (PS) in the alveolar fluid layer (AFL), which contributes to the local clearance both through hydrodynamic processes associated with Marangoni effects [76,77], and by the direct stimulation of alveolar macrophages [78,79]. It was proposed that a decrease in the surfactant concentration and its surface activity, observed due to PS adsorption on porous particles (with a large surface area), can slow down these natural defense mechanisms [80]. Both concepts, which can be adapted to increase the rate of drug delivery from aerosol drug carriers deposited in the RS, are schematically shown in Figure 7.

## 5. Selected Concepts and Methods of Improvement of Targeted Aerosol Delivery to Different Regions in the Respiratory System

When analyzing aerosol generation methods, it is clear that the ability to tailor particle size distribution is one of the most important ways to maximize the amount of drug targeted to different regions of the RS. This factor also determines possible side effects and drug losses. Therefore, this section discusses options for controlling particle size and maximizing inhalation dose, focusing mainly on nebulizers as aerosol delivery devices.

### 5.1. Nebulizer with a Valved Inhalation Chamber

**Figure 8:**
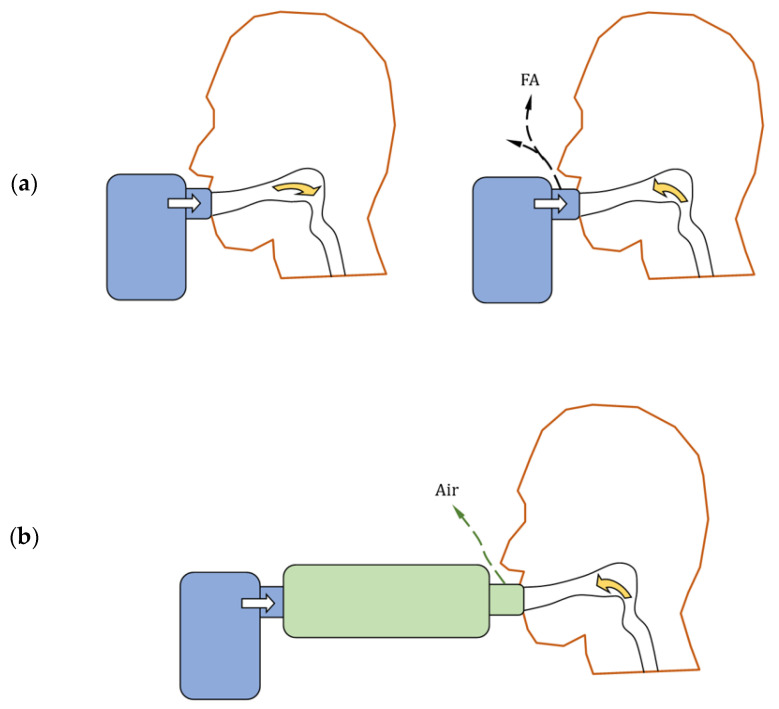
Aerosol transport from VMN during inhalation and exhalation (**a**) without IC (FA denotes fugitive aerosol) and (**b**) with valved IC (only air is exhaled).

In continuously operating (constant output) nebulizers, a part of the drug is lost as, so-called, fugitive aerosol, emitted during the exhalation phase, i.e., when it cannot be taken up by the patient [81]. To prevent this, the use of an inhalation chamber (IC), a simple device widely used with pMDI inhalers, has been proposed [82]. The IC connected to the nebulizer provides a holding space for the aerosol produced during exhalation and breath-hold, allowing it to be absorbed by the patient in the consecutive inhalation (Figure 8). Despite a loss of aerosol due to inertial and gravitational deposition in the IC, the effective availability of the drug to the patient is greater compared to the nebulizer without the chamber. This concept is particularly applicable to VMNs, which are typically not equipped with any valves which can minimize the fugitive emission.

The idea of using valved IC as a universal add-on device to any VMN was inspired by the Aerogen^®^ Ultra nebulizing system, in which the Aerogen^®^ Solo VMN was connected to an accessory that allowed this small VMN to be conveniently used as a handheld nebulizer [83,84]. Studies of the operation of the Intec Twister Mesh VMN nebulizer [85] (the nebulizer on the Polish market with a design analogous to Aura Portable Nebulizer available in the US [86]) connected to the valved IC showed that the amount of aerosol taken by the patient increases by almost 1.75-fold. In addition, the aerosol leaving the IC has a more favorable particle size distribution because the largest droplets are mainly deposited inside the chamber, allowing an increase in the fine particle fraction (*FPF*). The increase in the amount of aerosol delivered with droplets smaller than 5 μm is 2.8-fold when compared to an aerosol delivered without the valved IC [66]. A recent study with another VMN nebulizer (Intec Turbo Mesh [87]) for many drugs (glucocorticosteroids, anticholinergics, short- and long-acting β2-mimetics, SABA and LABA, and mucolytics) showed that the increase in the availability of fine droplets is 16% (SABA), 130–170% (glucocorticosteroids) and 190% (anticholinergics) [88]. Decreasing the mass of large droplets in inhaled aerosol should bring additional benefits to the patient, i.e., a reduction in local (oropharyngeal) side effects. Overall, it seems that the proposed concept is likely to improve the effectiveness of inhalation treatment and drug targeting to the lower RS using constant-output nebulizers, particularly VMNs.

### 5.2. Adjusting Aerosol Droplet Size via Physicochemical and Process Parameters of Nebulization

In general, the size of aerosol droplets formed in the nebulizer can be altered by the proper design of the atomizing system and adjustment of process conditions. The pressure of air used to atomize drugs in a particular model of a nebulizing system (compressor + dedicated nebulizing vessel) is constant. However, even for the constant pressure, there is a possibility to adjust the droplet size by tuning the inner geometry of the nebulizing vessel. A few commercially available jet nebulizers can produce droplets of different sizes after changing the internal geometry of the atomization system, e.g., by replacing a plastic element, which alters the distance between the nozzle and impaction baffle [89,90]. Increasing the diameter of the droplets allows us to increase the dose delivered to the upper parts of the RS, e.g., in the case of throat infections or croup (laryngotracheitis) [91]. 

Droplet size can also be modified by changing the physicochemical properties of the liquid, including the viscosity (or, more generally, the rheological properties), surface tension, and, sometimes, the ionic strength [92,93,94,95]. In the case of inhalation drugs, this can potentially be achieved by using proper (e.g., classified as GRAS—generally recognized as safe) drug additives, such as biosurfactants or viscosity modifiers of natural origin [96]. Such substances, for instance, biosurfactants, can be candidates for replacing synthetic adjuvants of inhalation drugs, such as Polysorbat 80 present in glucocorticosteroids for nebulization [97]. Several studies have reported that after thickening the aqueous solution (increase in viscosity), smaller aerosol droplets are generated in pneumatic nebulizers [92,93]. Despite a reduced emission efficiency (i.e., a lower delivery rate, hence a longer nebulization time) caused by an increase in liquid viscosity, the nebulized drug can be better targeted to the periphery of the lungs (smaller droplets mean a higher fine particle dose, FPD). Since some droplets can penetrate to the alveoli, it is also important to note that several proposed natural thickeners (sodium hyaluronate, xanthan gum, and agar) do not compromise PS function (in vitro studies [98]). The decrease in the mass median aerodynamic diameter (*MMAD*) of aerosol nebulized from liquids with higher viscosity may seem surprising, but it is easily explained based on an analysis of the aerosol formation process in a pneumatic nebulizer. A higher viscosity increases the droplet size produced by primary atomization in the two-fluid (pneumatic) nozzle causing a stronger impaction on the baffles and intensified secondary atomization, associated with the formation of fine droplets. This process is more intense for viscous liquids (larger primary droplets) than for liquids with lower viscosity; hence, a shift in the droplet size distribution toward smaller diameters (so an increase in FPD) is observed after increasing the viscosity.

This situation is different in ultrasonic nebulizers, both classical and VMNs. In the case of Newtonian liquids, the mass of very fine droplets decreases when viscosity increases, which, however, does not necessarily imply a decrease in the value of the volumetric median diameter *Dv50* (or *MMAD*), since a simultaneous decrease in the mode of the distribution is observed [99]. On the other hand, in the case of non-Newtonian fluids, additional submicron droplets appear in the aerosol, formed as satellites from the breakdown of droplets generated from the ultrasonic fountain, which results in a decrease in *Dv50*. Very viscous liquids are not atomized at all in ultrasonic nebulizers [93], which requires the use of only pneumatic devices for their nebulization. It has also been shown that in addition to shear (dynamic) viscosity, the dilatational (second) viscosity of the liquid also plays a role in droplet formation in ultrasonic devices [99].

Biosurfactants added to the nebulized solutions reduce the surface tension (similarly to other surfactants) and decrease the size of droplets emitted from pneumatic nebulizers due to the generation of a greater number of small droplets during primary atomization [100]. These droplets are small enough to avoid inertial collisions with the baffles of the nebulizing vessel and directly pass into the emitted aerosol. The resulting increase in the *FPF* allows for a better targeting of the lower RS. However, an undesirable effect of the presence of surface-active compounds is the foaming of the liquid in the nebulizing vessel [101]. It reduces the aerosol emission rate, since the nozzle is fed with a mixture of liquid and gas (i.e., foam) instead of liquid. Foaming also increases the so-called residual volume (the amount of liquid remaining inside the nebulizer at the end of nebulization), since the liquid is embedded in the foam that sticks to the walls of the vessel. Some reports of adding antifoaming agents to improve the nebulization of surface-active formulations are available in the literature [102,103].

The ionic strength of the solution, which can be adjusted by adding electrolytes, also affects the size of aerosol droplets, but only in VMN nebulizers with a metal membrane [94]. The reduction in the average droplet size, which also leads to an increase in *FPF* and a higher delivery rate, and which was observed for increased concentrations of electrolytes (e.g., NaCl, KCl, Na_2_SO_4_, MgCl_2_, LiCl, and other), was attributed to ionic interactions near the vibrating membrane, including the formation of an electrical double layer and the streaming potential [94,104]). These phenomena may have practical implications, e.g., for the nebulization of hypertonic saline. 

Physicochemical properties of the liquid also depend on temperature, which is, therefore, another parameter influencing the droplet size distribution and possibility of drug targeting in the RS. In all nebulizers, the change in the liquid temperature can be caused by thermal effects related to the operation of the nebulizer itself. The liquid is typically cooled in pneumatic devices due to adiabatic evaporation and is heated in classical ultrasonic nebulizers due to the energy provided from the vibrating piezoelectric crystal. It means that droplet size, and hence the efficiency of drug delivery, will be different at the beginning of inhalation and after several minutes of treatment [100]. The smallest changes in temperature, and thus minor effects on droplet size, are observed in VMN nebulizers. 

The evaporation of water (solvent) also increases the concentration of the sprayed solution in the systems where the liquid drains to the vessel (pneumatic and classic ultrasonic nebulizers). Both changes in temperature and concentration in time alter the size distribution of aerosol droplets released during a nebulization process, and this factor should also be taken into account when discussing the possibilities of targeting of inhaled drugs using nebulizers. The temperature can also be intentionally set to a higher level by thermostatting the nebulizer to obtain a desired droplet size distribution. For instance, it was shown that *Dv50* of nebulized saline in a selected nebulizer can be reduced from 5.23 μm at 20 °C to 4.23 μm by heating the liquid to 35 °C (thermo-aerosol) [105]. 

There is also another option for adjusting the droplet size of inhaled wet aerosol based on the thermodynamic effects in the aerosol phase. The mist released from the nebulizer can be diluted in the mouthpiece or mask, i.e., before entering the mouth, with intentionally added dry and warm air. This leads to a reduction in droplet size due to water evaporation and allows us to obtain a higher dose to potentially deliver to the lower RS. As confirmed by both CFD computations and experiments [106], the effect can be adjusted by air humidity and temperature, but also by the mixing ratio between the wet aerosol and the auxiliary air. This concept was also confirmed in invasive ventilation where dry air allowed us to reduce the *MMAD* of inhaled droplets from above 4 μm to below 2 μm, improving drug deposition in the lung periphery [107].

### 5.3. Targeted Drug Delivery by Functionalized Particles and Non-Conventional Therapeutics

In addition to the common techniques for delivering drugs by aerosol inhalation, several innovative concepts need to be noted. An interesting possibility for targeted drug delivery is the use of magnetic particles that can be attracted to disease areas in the lungs under the influence of an external magnetic field [108,109,110,111]. Despite the more complicated preparation of suitable drug carriers, this method offers a novel and attractive way of effective tumor targeting in lung cancer [110,112]. 

Several strategies that aimed to improve the targeting and effectiveness of drugs inhaled from DPIs were proposed using the functionalization of the surface of drug particles, which helps to increase both aerosolization and bioavailability. A recent overview by Knap et al. [113] indicated various approaches of formulating solid inhalable drug microparticles using polysaccharides (e.g., chitosan and hyaluronic acid), lipids (for instance, PS phospholipids such as dipalmitoyl phosphatidylcholine, DPPC, or dipalmitoyl phosphatidylglycerol, DPPG), proteins (e.g., fibroin), or polymers (such as poly(lactide-co-glycolide), PLGA, or polycaprolactone, PCL). The obtained effect on their bioavailability can include tuning the residence time on the lung surface and/or the rate of uptake by alveolar macrophages (AMs). For instance, a higher uptake of phospholipid-modified particles encapsulated in PLGA matrix by AMs was observed, in contrast to PEGylated particles [114]. Knowing that the effect is also dependent on the length of the polymeric chain, this strategy opens a possibility of adjusting the alveolar uptake and systemic effects of inhaled medicines [115]. Solid lipid particles (SLPs) are useful in the delivery of drugs poorly soluble in water (e.g., flavonoids such as quercetin, or glucocorticosteroids, such as budesonide) [111,116], and they also may offer a possibility for the temperature-responsive controlled release of encapsulated drugs [111]. Properties of powders for inhalation using DPIs can be improved by the addition of compounds such as amino acids (e.g., leucine [117,118]), proteins (e.g., lysozyme [119]), or PS components [120], which are admixed during powder preparation using various methods [48]. This allows us to obtain the optimal particle size distribution required to target the inhaled aerosolized drug to certain regions of the RS. Another interesting approach is focused on the preparation of nanostructured microparticles Few-micrometer nanostructured particles show good deposition in distal regions of the lungs and after hydration they can be transformed into nanoparticles that have a specific therapeutic function compared to larger particles which do not [119,121].

Aerosol therapy can be used not only with pharmaceuticals, but also other agents that show therapeutic effects in the lungs. For example, aerosol gene therapy has been proposed for several pulmonary diseases [122], including cystic fibrosis [123,124] and lung cancer [125,126]. The inhalation of aerosolized immunotherapeutics and monoclonal antibodies was also proposed in the anticancer therapy of the RS [125,127]. Inhaled bacteriophage aerosol has been considered as an alternative to inhaled antibiotics in pulmonary infections [128]. Some attempts have also been made in the field of inhalation of living cells [129], including the aerosol delivery of stem cells in COVID-19 treatment [130]. All these innovative concepts still require optimization of the aerosolization technique to ensure that sensitive biomolecules are not damaged due to the high stresses encountered during atomization.

### 5.4. Better Aerosol Targeting Using Electronic Technologies, Smart Inhalers, and Artificial Intelligence Solutions

A significant change in the effectiveness of aerosol therapy is expected from new electronically assisted solutions implemented in ‘smart inhalers’ [131,132]. Many of these devices are designed to help patients take their medications regularly and keep the patient and doctor informed about medication intake, which is essential for the proper control of therapy. Most noteworthy, however, are solutions that improve the handling and operation of the inhaler for optimal aerosol generation and inhalation, and hence a better controlled regional delivery of inhaled drugs. Some of these systems benefit from machine learning algorithms, resulting in the application of artificial intelligence (AI), which is expected to significantly improve the outcome of therapy [133]. 

Even before the era of smart inhalers, mechanical systems were developed, which were able to activate a pMDI only when the minimal inspiratory flow required to trigger a dose was achieved. For example, Easi-Breathe^®^ and Autohaler^®^ systems helped to eliminate coordination errors in pMDI inhalers [55,56]. Nowadays, the use of electronic sensors along with a digital analysis of the measured signals (pressure and airflow changes) allowed further advances in this field. As an example, the Adaptive Aerosol Delivery (ADD^®^) system is based on an analysis of the patient’s breathing pattern during nebulizer use and then an adjustment of aerosol release during only the first phase of inspiration, eliminating the problem of drug waste and fugitive emission [134]. The proper timing of aerosol release also allows us to better target the lower RS. Analogous sensor systems along with software were developed for DPI inhalers [135], where inhalation dynamics are known to be crucial for generating an aerosol dose with the desired PSD [106]. The number of smart devices and sensors currently available on the market is substantial (refer, e.g., to [136,137] for more comprehensive data), so only a few of the most popular systems are listed in Table 2. Most of them have a module enabling wireless communication with mobile phone applications, which makes them even more convenient for use by patients. Some systems focus on improving aerosol delivery to the lower part of the RS (e.g., Group 1), while others mainly serve to monitor the proper use of the device without analyzing factors affecting the actual aerosol delivery (e.g., sensors in Group 2). It can be expected that new capabilities of smart inhalers will be linked to the use of artificial intelligence (AI) algorithms for faster and better adjustment of optimal inhalation conditions based on the large amount of data collected from the population. 

## 6. Conclusions and Future Directions

The targeted delivery of drugs through the inhalation of aerosols is challenging. Its effectiveness is influenced by (i) the method of generation of the aerosol with suitable properties for penetration into a specific level of the respiratory system; (ii) individual patient characteristics (respiratory tract geometry and inhalation technique); and (iii) the patient’s ability to operate a given aerosol delivery device. It means that even when an aerosol with the desired properties is potentially available, its deposition in the areas requiring treatment is not ensured due to patient-related factors.

This paper discusses the key factors responsible for the targeted delivery of inhaled aerosol drugs, emphasizing both the importance of non-steadiness of airflow through the inhaler and respiratory airways, and the limitations in the prediction of deposition resulting from common simplifications in in silico and in vitro modeling. It was shown that other factors, such as the choice of drug delivery device and appropriate development of the properties/composition of the drug carrier particles, can affect the fate of inhaled therapeutic agents. 

Both physicochemical and physiological constraints prevent us from obtaining the perfect targeting of inhaled aerosols without drug losses. However, regional delivery can be optimized. Several possible strategies to improve the effectiveness of drug delivery to specific areas, such as the lower respiratory tract or nasal cavity, were highlighted. Some possible solutions are based on controlling aerosol droplet size generated in nebulizers by altering the physicochemical properties of liquid drugs, using the inhalation chamber as an add-on device, or diluting the aerosol with external air before its entrance into the RS. For aerosol directed into the nasal cavity, an attractive option is to use pulsed mist delivered from a nebulizer instead of using the rather ineffective delivery of large aerosol droplets sprayed from a nasal pump. Much is also expected from modern electronic systems implemented in smart inhalers, which are increasingly based on the use of artificial intelligence. The concepts presented in this paper indicate only some possible approaches to improving the targeted delivery of inhaled aerosol drugs, which still require further intensive research and the effective development of practical solutions.

## Figures and Tables

**Table 1:** Types of inhaling devices and systems for aerosol drug delivery to the lower airways.

Pressurized Metered Dose Inhalers (pMDIs)	Dry Powder Inhalers (DPIs)	Nebulizers	Other (Examples)
Hand-actuated	Single dose (capsule)	Pneumatic (jet)	Soft mist inhaler (Respimat^®^ SMI, Ingelheim Boehringer, Ingelheim am Rhein, Germany) [40]
Breath-actuated (mechanical) [55,56]	Containing pre-metered doses (on blister)	Ultrasonic - classic - vibrating mesh nebulizers (VMNs)	Thermal vaporization (sublimation/resublimation) breath actuated inhaler (Staccato^®^, Alexza Pharmaceuticals, Mountain View, CA, USA) [57]
They can be used both with or without holding chamber (or spacer)	Metering the dose from powder reservoir		

**Table 2:** Examples of smart inhalers and inhalation systems.

Name of the System	Basic Characteristics	‘Smart’ Functions
**Group 1: Nebulizers and fine mist inhalers**		
I-neb^®^ AAD^®^ (Adaptive Aerosol Delivery) [138]	Hand-held VMN system with electronic interface which measures pressure changes in the airflow	The system sets the duration of aerosol pulse to deliver the drug only in the first phase of inspiration by continuously adapting to changes in breathing pattern. It maximizes the delivered dose and minimizes drug losses [134].
Breelib™ [139]	Breath-activated VMN	The system controls the flow and inhaled volume to maximize the dose delivered to deep lung regions.
Akita^®^ Jet [140]	Electronic system used to optimize drug delivery from the jet nebulizer	The system guides the patient to inhale with a required inspiratory flow rate and time, increasing the targeted drug delivery to deep lung regions [141].
AERx^®^	Fine mist aerosol inhaler with breath control technology. Drug dose is delivered one or two breaths from single-use AERx Strip	The system releases aerosol during the first phase of inhalation and controls the inhalation flow, increasing the targeted drug delivery to deep lung regions. The system with AERx Strip may be a platform for personalized aerosol therapy [142,143].
**Group 2: Attachable (add-on) devices/accessories/sensors for pMDIs and DPIs**		
Hailie^®^ [144]	Electronic attachable sensor for several models of pMDI or DPI	The sensor records time/date each actuation of pMDI or DPI. Some versions may also detect inhaler shaking prior to use and flow during inhalation.
FindAir^®^ One [145]	Electronic module attached to the top of pMDI or on some DPIs	The sensor transmits the information regarding the inhaler use to the mobile phone application.
Propeller^®^ [146]	Electronic module attached to the top of pMDI	The sensor detects and records the time of actuation of pMDIs or DPIs.
Capmedic^®^ [147]	Electronic module attached to the top of pMDI	The sensor records shaking, the upright position of pMDI, proper timing of the actuation, the and duration of inhalation. It also records the time of pMDI actuation.
Respiro^®^ [148]	Electronic attachable sensor	The sensor records the actuation and flow of inhalation in DPIs or pMDIs.
**Group 3: DPIs—built-in systems**		
Digihaler^®^ [149]	Electronic built-in sensor	The sensor records each actuation and the flow of inhalation.

## Data Availability

The data presented in this study are available in this article.
